# γH2A/γH2AX Mediates DNA Damage-Specific Control of Checkpoint Signaling in *Saccharomyces cerevisiae*

**DOI:** 10.3390/ijms25052462

**Published:** 2024-02-20

**Authors:** Jasmine Siler, Na Guo, Zhengfeng Liu, Yuhua Qin, Xin Bi

**Affiliations:** 1 Department of Biology, University of Rochester, Rochester, NY 14627, USA; 2 College of Food Science and Engineering, Jilin University, Changchun 130012, China

**Keywords:** camptothecin, DNA damage checkpoint, γH2A/γH2AX, Rad9, Rad53

## Abstract

DNA lesions trigger DNA damage checkpoint (DDC) signaling which arrests cell cycle progression and promotes DNA damage repair. In *Saccharomyces cerevisiae*, phosphorylation of histone H2A (γH2A, equivalent to γH2AX in mammals) is an early chromatin mark induced by DNA damage that is recognized by a group of DDC and DNA repair factors. We find that γH2A negatively regulates the G2/M checkpoint in response to the genotoxin camptothecin, which is a DNA topoisomerase I poison. γH2A also suppresses DDC signaling induced by the DNA alkylating agent methyl methanesulfonate. These results differ from prior findings, which demonstrate positive or no roles of γH2A in DDC in response to other DNA damaging agents such as phleomycin and ionizing radiation, which suggest that γH2A has DNA damage-specific effects on DDC signaling. We also find evidence supporting the notion that γH2A regulates DDC signaling by mediating the competitive recruitment of the DDC mediator Rad9 and the DNA repair factor Rtt107 to DNA lesions. We propose that γH2A/γH2AX serves to create a dynamic balance between DDC and DNA repair that is influenced by the nature of DNA damage.

## 1. Introduction

**Figure 1:**
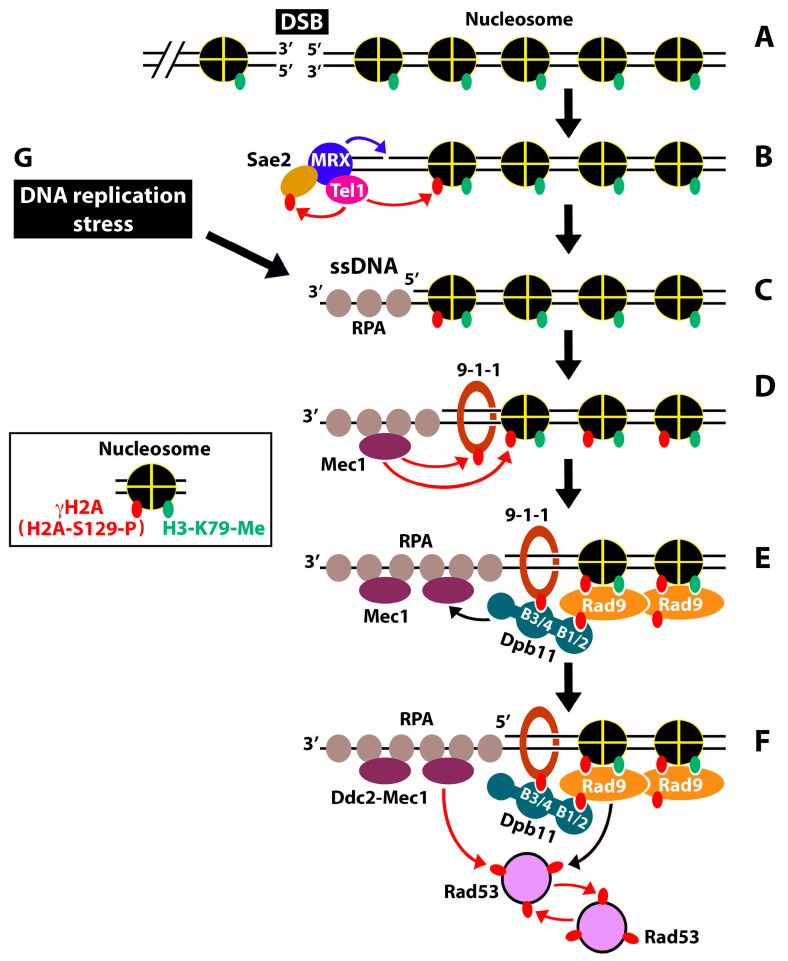
Mechanism of DNA damage-checkpoint signaling in *S. cerevisiae*. Red arrows denote phosphorylation. Black arrows denote recruitment and/or activation. Phosphorylation of Ser129 of histone H2A and methylation of lysine 79 of histone H3 on the nucleosome are indicated in the inset. (**A**) A DNA DSB in the context of chromatin. (**B**–**F**) Events of DDC in response to a DNA DSB. See the text for descriptions. (**G**) DNA replication stress.

Genotoxins cause genome instability by damaging DNA and/or blocking DNA replication. Cells have evolved intricate mechanisms for safeguarding genome integrity that are collectively called DNA damage response [1]. DNA damage response recognizes DNA lesions, activates cell checkpoints to arrest cell cycle progression, stabilizes DNA replication forks during the S phase, and promotes DNA damage repair [1,2]. The coordination of various activities of DNA damage response depends on a cascade of protein phosphorylation carried out by a series of apical and effector kinases [3]. In *Saccharomyces cerevisiae*, the apical DNA damage kinases are Mec1 and Tel1, homologs of mammalian ATR and ATM, respectively [4,5,6]. They are recruited to the ends of double-stranded DNA breaks (DSBs) or single-stranded DNA (ssDNA), resulting from DNA damage or replicative stress [7,8]. DSBs are recognized by the MRX complex (consisting of Mre11, Rad50, and Xrs2), together with Sae2 [9] (Figure 1B). MRX recruits Tel1 and activates its kinase activity [9,10,11]. Tel1 phosphorylates histone H2A at serine 129 (equivalent to serine 139 of the histone variant H2AX in mammals), creating H2A-S129-P, or γH2A (equivalent to mammalian γH2AX), containing nucleosomes [3,12] (see inset in Figure 1). Note that histone H2A-S129 in the chromatin surrounding DSBs is also subject to phosphorylation by Mec1 later during DDC signaling [7,12,13,14] (Figure 1D). γH2A/γH2AX spreads up to 50 kb on either side of a DSB [15].

MRX has both endonuclease and exonuclease activities and helps to recruit exonuclease Exo1 [16,17,18]. The combined action of nuclease activities of MRX and Exo1 mediates the initial resection of the 5′ strand of a DSB, leading to a short 3′ ssDNA overhang covered by the ssDNA binding complex RPA (Figure 1C). The DNA damage clamp 9-1-1 binds the junction between the dsDNA and 5′ ssDNA [8] (Figure 1D). 9-1-1 and ssDNA-RPA activate the Exo1 and Dna2-STR nucleases, allowing them to perform more extensive (long-range) DNA end resections [3,8].

The generation of the 3′ ssDNA triggers the dissociation of Tel1 from DSBs, while promoting the activation of Mec1-dependent signaling [7,19]. Mec1 (in complex with Ddc2) is recruited to DSBs by binding RPA-associated ssDNA [7,8] (Figure 1D). Mec1 phosphorylates 9-1-1 and histone H2A (which maintains and expands γH2A-containing chromatin) [7,12,13,14,20] (Figure 1D). The phosphorylated 9-1-1 recruits the scaffold protein Dpb11 [21,22,23]. Dpb11 functions in DNA damage signaling by activating Mec1 and by helping to recruit the DDC adaptor/mediator kinase Rad9 [21,24,25] (Figure 1E). Dpb11 bears four BRCT domains (referred to as B1 to B4 here) that interact with phosphoproteins. Dpb11 binds the phosphorylated 9-1-1 via its BRCT motifs 3+4 (B3/4), and the phosphorylated Rad9 via B1/2 (Figure 1E). Besides binding Dpb11, Rad9 also recognizes γH2A and the methylated lysine 79 of histone H3 (H3-K79-me) [26,27] (Figure 1E). Note that whereas the formation of γH2A is induced by DNA damage, histone H3-K79 methylation by Dot1 occurs independently of DNA damage [27].

Upon activation via Mec1-dependent phosphorylation, Rad9 binds and activates the checkpoint effector kinase Rad53 that then undergoes intermolecular autophosphorylation [28,29,30,31] (Figure 1F). Rad53 is also phosphorylated by Mec1 [31,32] (Figure 1F). Mec1 and Tel1 also phosphorylate and activate the Chk1 kinase (not shown in Figure 1) [33]. The activated Rad53 and Chk1 molecules are then released from the sites of DNA damage to transduce signals to downstream targets as part of a signaling cascade, leading to cell cycle arrest and DNA repair [3,8]. The above-described DNA damage signaling critically requires the ssDNA and dsDNA/ssDNA junction, which results from DSB end resections (Figure 1D–F). Note that ssDNA gaps may also be generated at stalled replication forks during replicative stress (Figure 1, G→C), which would potentially also trigger Rad9-dependent DDC signaling, similar to the ssDNA generated by DSB end resections [3]. Stalled replication forks also induce DNA replication checkpoint signaling, which is dependent on Mrc1 instead of Rad9 [3,34,35].

In addition to inducing checkpoint responses, DNA damage-signaling kinases also control DNA repair by helping the recruitment and/or function of repair factors [3]. For example, the recruitment of the Slx4/Rtt107 complex involved in homologous recombination (HR) repair to DNA lesions is promoted by the phosphorylation of Slx4 and histone H2A. Specifically, the phosphorylation of Slx4 mediates its interaction with Dpb11 associated with 9-1-1 at the 5′ recessed DNA junction, whereas γH2A is recognized by Rtt107 [36,37,38]. As Dpb11 and γH2A also mediate the recruitment of Rad9, Rad9 and Slx4/Rtt107 may compete to bind to DNA lesions, leading to a competitive relationship between DNA-damage signaling and repair processes. Consistently, it has been shown that the deletion of Rtt107 results in enhanced checkpoint signaling induced by genotoxins [39]. We previously found that the deletion of Fun30, a chromatin remodeler involved in the HR repair of DSBs, also enhanced DDC signaling induced by the genotoxin camptothecin (CPT) or methyl methanesulfonate (MMS) [40]. CPT is a DNA topoisomerase I (Top1) poison that traps Top1 when it is crosslinked to DNA during its enzymatic function of relaxing DNA supercoiling [41]. The ternary complex of CPT-Topo I-DNA is a dangerous lesion, as it may block DNA replication in the S phase and may generate DSBs when it falls apart, triggering DDC activation [41]. MMS alkylates DNA and stalls progressing the DNA replication fork, which may lead to the formation of DSBs and/or ssDNA that induce checkpoint responses [8].

While investigating how Fun30 impacts DDC signaling, we discovered that, interestingly, blocking H2A-S129 phosphorylation (γH2A) enhances the CPT-induced phosphorylation of Rad53 and Rad9, which points to a negative impact of γH2A on DDC signaling. We showed that γH2A suppresses the G2/M checkpoint in response to CPT. Moreover, we found that γH2A also negatively regulates the DDC induced by MMS. However, prior work has demonstrated positive or no roles of γH2A in DDC in response to other DNA-damaging agents, such as ionizing radiation and the radiomimetic agent phleomycin that can cause single or double-stranded DNA breaks [42]. We propose that γH2A mediates a dynamic balance between DDCs and DNA repair that is influenced by the nature of DNA damage.

## 2. Results

### 2.1. γH2A Negatively Regulates DNA Damage Signaling

**Figure 2:**
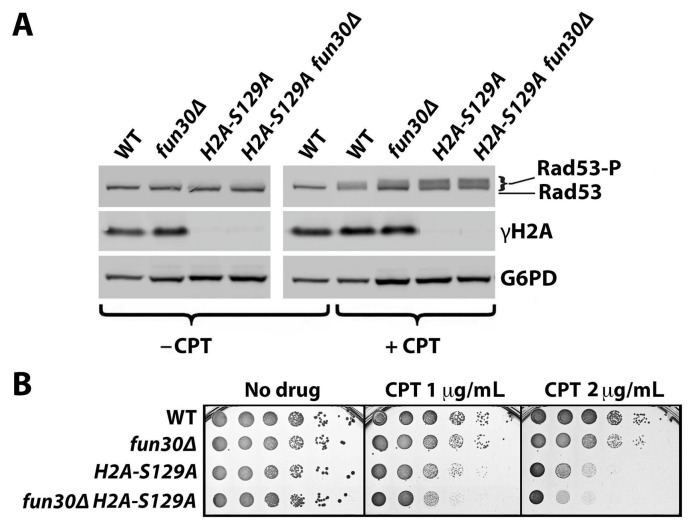
γH2A inhibits DDC signaling and promotes CPT resistance independently of Fun30. (**A**) Western blot analysis of Rad53, γH2A, and G6PD (with G6PD, glucose-6-phosphate dehydrogenase, as a loading control) from indicated strains (#1–4 in Table 1) with (+CPT) or without (−CPT) CPT treatment. Exponentially growing cells were incubated at 30 °C with or without CPT at 5 μg/mL for 90 min. Protein extracts from these cells were subjected to SDS-PAGE and Western blotting, followed by the detection of Rad53, γH2A, and G6PD with respective antibodies. Rad53-P, phosphorylated Rad53. (**B**) Growth phenotypes of serial dilutions of indicated strains (#1–4 in Table 1) on media with or without CPT after incubation at 30 °C for three nights.

In a previous study, we found that the Fun30 chromatin remodeler negatively impacts DDC, as Fun30 deletion increases the DNA damage signaling reflected by the phosphorylation of Rad53 [40]. Histone H2A-S129 phosphorylation, or γH2A (equivalent to γH2AX in mammals), has been shown to hinder Fun30 binding to nucleosomes in vitro [43]. It was, therefore, reasonable to propose that γH2A promotes DDC by negatively regulating Fun30 binding to chromatin, and, consequentially, γH2A’s role in DDC would be dependent on Fun30. To test this hypothesis, we examined if *hta-S129A* (with the histone H2A-S129 changed to a *nonphosphorylable* alanine) fails to promote DDC signaling when Fun30 is absent. Specifically, we monitored the CPT-induced Rad53 phosphorylation in *hta-S129A* and *fun30Δ* single and double mutants as well as WT cells. The level of Rad53 phosphorylation in each strain was reflected by the abundance of phosphorylated Rad53 (Rad53-P) relative to that of unphosphorylated Rad53 detected by SDS-PAGE and Western blotting (Figure 2A).

Surprisingly, we found that the Rad53-P level in the *hta-S129A fun30Δ* double mutant was higher than that in the *fun30Δ* cells (Figure 2A), indicating that γH2A negatively impacts DNA damage signaling in the absence of Fun30. As a matter of fact, the Rad53-P level in the *hta-S129A* mutant was also higher than that of wild type (WT) cells (Figure 2A). This was corroborated by a similar finding in hta-S129* vs. wild type strains. H2A-S129* is a truncated allele of histone H2A that is deleted for its C-terminal four amino acids, including S129 [12]. The above observations argue against the notion that γH2A *promotes* DDC signaling and instead indicate that γH2A, similar to Fun30, *negatively impacts* DDC signaling in yeast. The negative impact of γH2A on Rad53-P persisted in the absence of Fun30 (Figure 2A), suggesting that γH2A regulates DDC signaling independently of Fun30. A CPT-sensitivity test of the *hta-S129A* and *fun30Δ* single and double mutants revealed that γH2A plays a larger role than Fun30 in CPT-resistance, which is independent of Fun30 (Figure 2B).

### 2.2. γH2A Controls CPT-Induced G2/M Checkpoint

**Figure 3:**
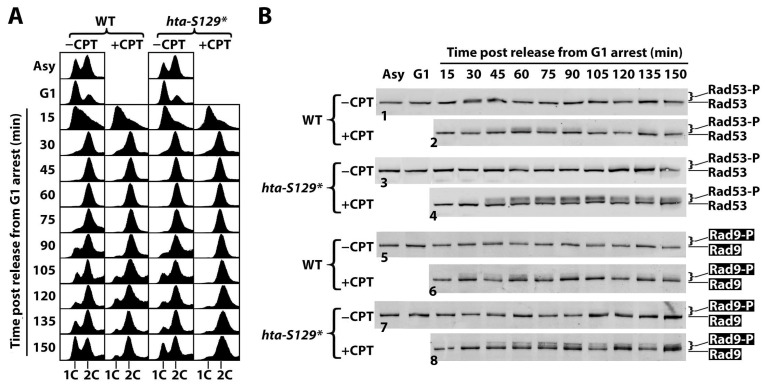
γH2A hinders G2/M checkpoint in response to CPT. Asynchronous cells of the WT and hta-S129* strains (#7 and 8 in Table 1) were arrested in G1 by α-factor and released into medium with (+CPT) or without (−CPT) 5 μg/mL of CPT and were incubated for 150 min. Aliquots of cells were collected at the indicated time points for FACS analysis (**A**) and protein extraction for examining Rad53 and HA-tagged Rad9 by SDS-PAGE and Western blotting (**B**). Note 1C and 2C in (**A**) indicate one and two copies of chromosomal DNA, respectively. In (**B**), panels 1 and 2 designate Rad53 Western blotting results from wild type cells (WT) treated without (−CPT) or with (+CPT) CPT, as indicated. Panels 3 and 4 designate Rad53 results from hta-S129* cells treated without or with CPT. Panels 5 and 6 designate Rad9-HA Western blotting results from wild type cells (WT) treated without or with CPT. Panels 7 and 8 designate Rad9-HA results from hta-S129* cells treated without or with CPT.

In the above experiments, Rad53-P was measured in exponentially growing cells treated with CPT for 90 min. The increase in Rad53-P, caused by the lack of γH2A, might reflect a heightened intra-S or G2/M checkpoint. To test these possibilities, we arrested hta-S129* and WT cells in G1 with α-factor and then released them into a fresh medium with or without CPT for a further 150 min of incubation. Aliquots of the culture were taken every 15 min after G1 release to monitor cell cycle progression and DNA damage signaling by FACS and Western blotting, respectively. The FACS data show that the WT cells reached G2 about 45 min after G1 release and started to exit mitosis at about 75 min in the absence of CPT (Figure 3A, WT, −CPT panel). At 150 min, the culture consisted of significant proportions of cells in the G1, S, and G2/M phases, which is similar to an asynchronous cell culture (Figure 3A, WT, −CPT panel), likely reflecting a loss of synchrony in cell-cycle progression after prolonged incubation. The presence of CPT had no effect on the progression of the WT cells from G1 to G2 (up to ~60 min after the release from G1 arrest) (Figure 3A, WT; compare the −CPT and +CPT panels), demonstrating that CPT does not induce an intra-S checkpoint. On the other hand, only a small portion of cells were able to exit mitosis after further incubation in the presence of CPT (Figure 3A, WT, +CPT panel, 75–150 min), indicating that CPT triggered a G2/M checkpoint response. The progression of the hta-S129* cells in the cell cycle following G1 release in the absence of CPT was similar to that of the WT cells (Figure 3A; compare the WT and hta-S129* −CPT panels), suggesting that γH2A is not required for normal cell proliferation. In the presence of CPT, hta-S129* did not delay S phase progression (Figure 3A; compare the WT+CPT and hta-S129* +CPT panels; 15–60 min) but caused a tighter blockage of the exit from mitosis (Figure 3A, compare the WT+CPT and hta-S129* +CPT panels; 75–150 min). These results indicate that hta-S129* enhances the CPT-induced G2/M checkpoint.

Consistent with the above FACS results which show an enhancement of the CPT-induced G2/M cell cycle checkpoint by hta-S129* mutation, we found that hta-S129* increased the level of the CPT-induced Rad53-P when cells entered the G2 phase (Figure 3B; compare panels 2 and 4). This was accompanied by an increase in the phosphorylation of the DDC adaptor Rad9 (Rad9-P) (Figure 3B; compare panels 6 and 8). Therefore, γH2A downregulates the activation of CPT-induced DDC signaling. It is noteworthy that in the WT cells, the CPT-induced Rad53-P and Rad9-P peaked around 60 min when the WT cells entered G2/M and declined afterwards (Figure 3B, panels 2 and 6). On the other hand, in the hta-S129* cells, the Rad53-P and Rad9-P levels stayed high after reaching their maximum values between 60 and 75 min (Figure 3B, panels 4 and 8). These results suggest that γH2A also negatively regulates the *recovery* of the G2/M checkpoint induced by CPT.

### 2.3. γH2A May Downregulate DDCs by Hindering Rad9 Recruitment to DNA Lesions

**Figure 4:**
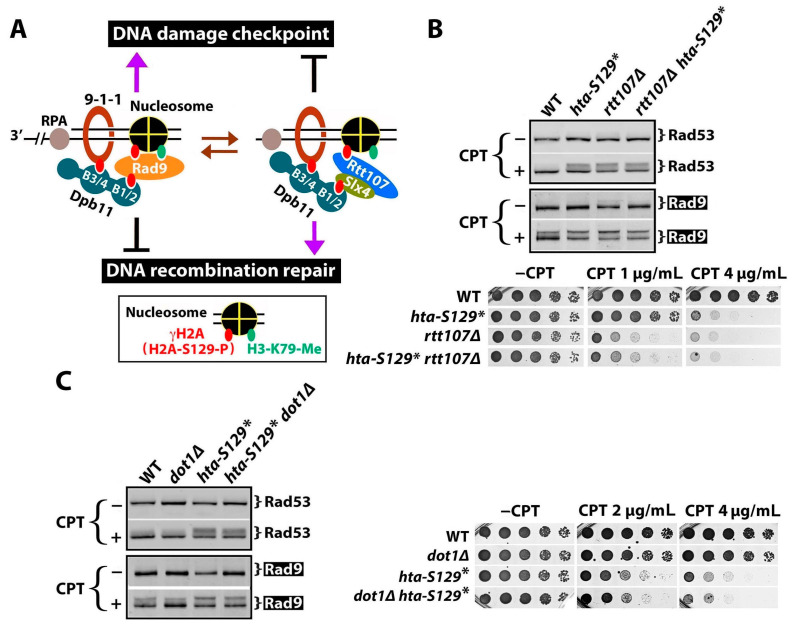
γH2A may downregulate DDC by hindering Rad9 recruitment to DNA lesions. (**A**) Model for negative regulation of DDC by γH2A. See the text for descriptions. (**B**,**C**) Results from Western blot analysis of Rad53 and Rad9-HA from indicated strains with or without CPT treatment at 5 μg/mL for 90 min, as well as growth phenotypes of the strains on media with or without CPT. Strains tested are #5, 6, 9, and 10 (Table 1) in (**B**) and #5, 11, 6, and 12 in (**C**).

Our finding that γH2A negatively impacts CPT-induced DDC signaling (Figure 2 and Figure 3) is apparently counterintuitive, as γH2A is known to help recruit Rad9 to chromatin (Figure 1E) [26]. This *conundrum* might stem from the competition between Rad9 and the DNA repair scaffold Slx4/Rtt107 for binding γH2A and Dpb11 at DNA damage sites [22,36,37,38,45,46]. Rad9 bears a double BRCT domain that recognizes γH2A, and Rad9 phosphorylated at S464 and T474 by CDK binds to the B1/2 domain of the Dpb11 tethered to the 9-1-1 complex [26,46] (Figure 4A, left). On the other hand, the BRCT domain of Rtt107 also recognizes γH2A, and Slx4 phosphorylated by CDK also binds to the B1/2 domain of Dpb11 [36,45,46] (Figure 4A, right). The competition between Rad9 and Slx4/Rtt107 for binding γH2A and Dpb11 is thought to yield a dynamic balance between DNA lesions associated with Rad9 and those with Slx4/Rtt107 [39] (Figure 4A). In line with this model, the deletion of Rtt107 has been shown to increase DDC signaling, likely by allowing Rad9 to bind γH2A and Dpb11 more efficiently [39] (Figure S1B). A loss of γH2A (as in hta-S129* or *hta-S129A* mutant) would eliminate the γH2A-mediated recruitment of Rad9 and Slx4/Rtt107 to damaged chromatins, but Rad9 and Slx4/Rtt107 can still interact with Dpb11, and Rad9 can additionally associate with H3-K79-me (Figure S1C). We propose that under this circumstance of the hta-S129* mutant, Rad9 outcompetes Slx4/Rtt107 for engaging DNA lesions (Figure S1C), resulting in an increase in DDC signaling, as was shown in Figure 2A.

In the above model, the negative role of γH2A in DDC is mediated by its association with Rtt107 to damaged chromatin (Figure 4A, right). Consistently, we show that γH2A no longer negatively impacts Rad9-P or Rda53-P in the absence of Rtt107 (Figure 4B, top; compare the *rtt107Δ* and rtt107Δ hta-S129* in the +CPT panel). Moreover, γH2A plays a smaller role in cellular resistance to CPT than Rtt107, and this role is lost in the absence of Rtt107 (Figure 4B, bottom). These results support the notion that γH2A regulates yeast response to CPT-induced DNA lesions via recruiting Rtt107 to damaged loci.

Unlike γH2A, which can interact with both Rad9 and Rtt107, histone-H3-K79 methylation (H3-K79-me) is recognized by Rad9, but not Rtt107 (Figure 4A), and should, therefore, play a positive role in DDC. Consistently, H3-K79-me or Dot1, responsible for H3-K79 methylation, has been shown to aid in DDC induced by phleomycin or ionizing radiation (IR) [27,44,47,48]. We found here that *dot1Δ* reduces CPT-induced Rad9-P (Figure 4C, left). The CPT-induced Rad53-P level in the *dot1Δ* cells was similar to that in the WT (*DOT1*) cells (Figure 4C, left), possibly because only a limited amount of Rad53-P was induced by CPT in the WT cells to begin with (Figure 4C). In the hta-S129* cells, *dot1Δ* moderately reduced the CPT-induced Rad9-P and Rad53-P (Figure 4C), which is consistent with the notion that H3-K79-me assists Rad9 recruitment to chromatins independently of γH2A. Note that Rad9-P and Rad53-P in the hta-S129* dot1Δ double mutant were more robust than those in the *dot1Δ* and WT cells (Figure 4C), suggesting that blocking Rad9 recruitment via association with chromatin marks (γH2A and H3-K79-me) does not markedly reduce DDC signaling. This is consistent with the notion that DDC signaling can proceed via the Dpb11-mediated recruitment of Rad9 independently of the chromatin (γH2A and H3-K79-me)-dependent pathway of Rad9 recruitment [44] (Figure S1F). Although Dot1 contributes to DDCs, it has little or no effect on CPT resistance in the presence or absence of γH2A (Figure 4C, right).

### 2.4. The Effect of γH2A on Checkpoint Signaling Is DNA Damage-Dependent

That γH2A exhibits a negative effect on the CPT-induced G2/M checkpoint (Figure 2 and Figure 3) is not in line with previous studies which suggest positive or no functions of γH2A in DDC [26,44,48]. For example, γH2A was found to contribute to the G1 checkpoint but not the G2/M checkpoint induced by IR or phleomycin [26,44,48]. Moreover, γH2A was shown to play a minor role in the intra-S checkpoint induced by MMS [26,44,48]. Our work differs from these studies in the genotoxin used (CPT vs. IR, phleomycin or MMS) and the genetic backgrounds of the yeast cells used, which may influence the DDC responses examined [8]. In an attempt to explain the apparent discrepancies between our and others’ studies, we systematically tested the effects of hta-S129* on DDCs in response to MMS and phleomycin in addition to CPT in the same genetic background of JKM139 (Table 1). Both Rad53-P and Rad9-P were measured as indicators of DDC signaling in exponentially growing cells. The effects of deleting the known DDC regulators Slx4, Rtt107, and Sae2 on DDC signaling were also monitored as controls.

**Figure 5:**
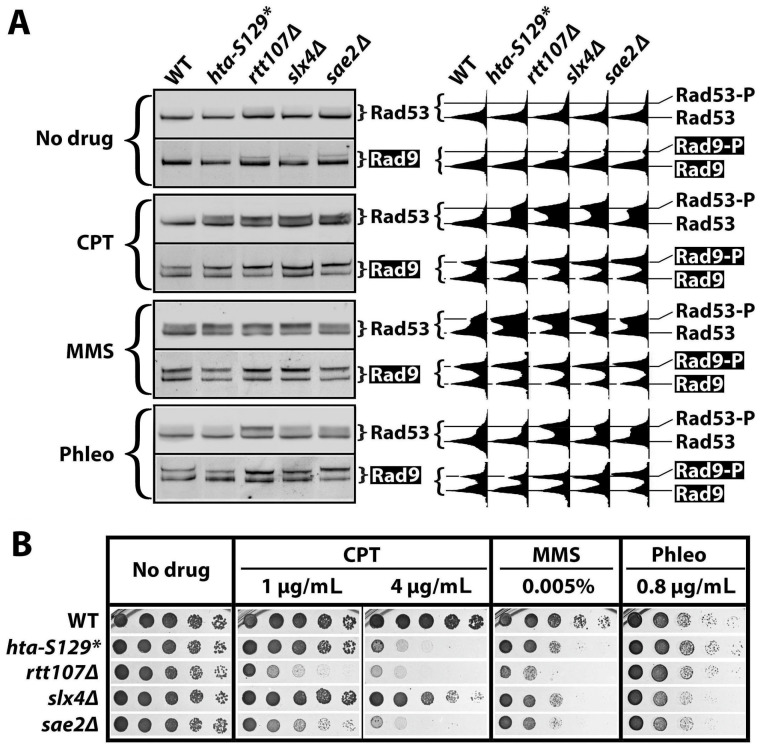
γH2A regulates DDC signaling in a DNA damage-specific manner. (**A**) Western blot analyses of Rad53 and Rad9-HA (Rad9) from indicated strains (#5, 6, 9, 13, and 14 in Table 1) that were treated with CPT, MMS, or phleomycin (Phleo) or that were mock treated (No drug). Exponentially growing cells of each strain were treated for 90 min with 5 μg/mL of CPT, 0.01% MMS, or 5 μg/mL of phleomycin. Bands and smears corresponding to Rad53, Rad53-P, Rad9, and Rad9-P in the blots were quantified/scanned using NIH ImageJ software version 2.0.0-rc-43/1.52n (National Institute of Health, Bethesda, MD, USA) and the profiles are displayed to the right of the blot images. (**B**) Growth phenotypes of strains #5, 6, 9, 13, and 14 (Table 1) on media with or without the indicated genotoxins.

As shown in Figure 5A, htaS-129A*, *slx4Δ*, *rtt107Δ*, and *sae2Δ* increased the CPT-induced Rad53-P and Rad9-P to various degrees (compare the CPT and WT panels), confirming that γH2A, Slx4, Rtt107, and Sae2 all downregulate DDC in response to CPT. The MMS induced robust Rad53-P and Rad9-P in wild cells (Figure 5A, MMS panel). The hta-S129* mutation increased the MMS-induced Rad53-P and, to a lesser extent, Rad9-P (Figure 5A), indicating that γH2A also negatively impacts DDC signaling in response to MMS. Consistent with previous studies, *rtt107Δ*, *slx4Δ*, and *sae2Δ* were all found to markedly increase the MMS-induced Rad53-P (Figure 5A) [39,49]. We show here that these mutations also enhanced Rad9-P in response to MMS (Figure 5A).

Phleomycin induced a moderate level of Rad53-P and a relatively higher level of Rad9-P (Figure 5A, Phleo panel). The hta-S129* mutation moderately reduced the phleomycin-induced Rad53-P and Rad9-P (Figure 5A). On the other hand, the *rtt107Δ*, *slx4Δ*, and *sae2Δ* mutations all increased both Rad53-P and Rad9-P in response to phleomycin (Figure 5A). Therefore, phleomycin-induced DDC signaling is partially dependent on γH2A and is inhibited by Slx4/Rtt107 and Sae2.

The results from the above experiment reveal that γH2A plays a negative role in checkpoint signaling induced by CPT or MMS but a positive role in checkpoint induced by phleomycin. This was corroborated by data obtained from independent experiments on the effects of *hta-S129A* on DDC signaling in two genetic backgrounds (Figure S2). Therefore, γH2A exhibits DNA damage-specific effects on DDC signaling, which is in contrast to Slx4, Rtt107, or Sae2 which dampens checkpoint induced by each DNA damage tested above.

We also examined how hta-S129* as well the as *slx4Δ*, *rtt107Δ*, and *sae2Δ* mutations affect cell survival in the presence of CPT, MMS, or phleomycin. As shown in Figure 5B, each mutation decreased cellular resistance to all three genotoxins to various degrees, except for hta-S129* and *rtt107Δ*, which did not have a significant effect on phleomycin resistance. The latter is interesting since hta-S129* and *rtt107Δ* had opposite effects on phleomycin-induced DDC signaling (Figure 5A). On the other hand, the *slx4Δ* and *sae2Δ* mutants had similar levels of DDC signaling in the presence of CPT (Figure 5A) but exhibited drastically different degrees of CPT resistance (Figure 5B). These results demonstrate the lack of a direct correlation between the degree of DDC signaling and cell survival in the presence of DNA damage.

## 3. Discussion

In this report, we show that yeast γH2A negatively impacts the CPT-induced G2/M checkpoint and downregulates MMS-induced DDC signaling. On the other hand, γH2A plays a positive role in DDC in response to phleomycin. γH2A participates in DNA damage response by serving as a docking site for multiple DDC or DNA repair factors, including Rad9 and Slx4/Rtt107 (see Figure 1E and Figure 4A). Given the mutually exclusive nature of γH2A association with these factors, γH2A has the potential to positively or negatively regulate various processes involved in DNA damage response. For example, γH2A can serve dual, opposing functions in DDC activation. One is to promote DDC by recruiting Rad9 to DNA lesions, and the other is to inhibit DDC by recruiting Slx4/Rtt107 [39] (Figure 4A). The outcome of the competition between these two functions would determine whether γH2A has a net positive or negative impact on DDC. As such, our finding that blocking γH2A increases DDC signaling induced by CPT or MMS (Figure 5 and Figure S2), which likely reflects that the γH2A-mediated recruitment of Slx4/Rtt107 outweighs Rad9 recruitment in the presence of DNA lesions induced by CPT or MMS. On the other hand, Rad9 recruitment by γH2A seems to prevail over Slx4/Rtt107 recruitment in response to phleomycin or IR, as the lack of γH2A reduces phleomycin- or IR-induced DDC [26,44] (Figure 5 and Figure S2).

That γH2A differentially impacts DDCs in response to CPT, MMS, phleomycin, or IR can be explained by assuming that the result of the competition between Rad9 and Slx4/Rtt107 for binding γH2A at a DNA lesion is dependent on the nature/type of the lesion. Both CPT and MMS trigger DDCs by inducing replicative stress in the S phase, whereas phleomycin and IR can induce DSBs and DDCs in any phase of the cell cycle. It is tempting to propose that a DNA replication stress-induced DNA lesion presents a more favorable context for the Slx4/Rtt107–γH2A interaction than the Rad9–γH2A interaction, whereas a DSB induced by phleomycin or IR is more favorable for the Rad9–γH2A interaction.

It is noteworthy that although CPT and MMS can each trigger a G2/M checkpoint [40,50,51] (Figure 2), checkpoint activation requires the host cell to traverse the S phase [40,50]. The CPT-trapped Top1 cleavage complex or the MMS-mediated DNA methylation stalls DNA replication in the S phase, which has to be resolved to allow for the completion of DNA replication. Mechanisms for the restart of DNA replication may involve the generation of altered fork structures (as fork restart intermediates) harboring ssDNA gaps that can trigger DDCs [52]. We imagine that these distorted fork structures are different from resected (simple) DSB ends induced by phleomycin or IR in influencing γH2A interactions with Rad9 and/or Slx4/Rtt107. Specifically, we posit that Slx4/Rtt107 outcompetes Rad9 for binding γH2A within the fork restart intermediates induced by CPT or MMS, whereas Rad9 is favored for binding γH2A at resected DSBs in the presence of phleomycin or IR. Consequentially, γH2A plays a net negative role in DDC signaling in response to CPT or MMS but a positive role in DDCs triggered by phleomycin or IR.

Cells defective in proper DDC signaling are usually sensitive to genotoxins [1]. However, our survey of DDC signaling and CPT sensitivities of the series of mutants described in this work revealed a lack of a causative link between the level of DDC signaling and the degree of CPT resistance, which is line with prior observations [33,53,54]. This could be because efficient cellular resistance to a genotoxin requires an “optimal” level of DDC signaling. Alternatively, or in addition, genotoxin resistance may reflect aggregated effects of the genotoxin on different aspects of cellular response to DNA damage. Given that many factors involved in DNA damage response have multiple functions that are not restricted to DDCs, the deletion of a particular factor may have effects on not only DDCs but also DNA replication and/or DNA repair in the presence of a genotoxin. The combination of these effects likely determines the ability of the mutant to withstand DNA damage induced by the genotoxin. The separation-of-function mutations of DDC factors would be particularly useful for examining how these factors contribute to DCC signaling and genotoxin resistance.

## 4. Materials and Methods

### 4.1. Yeast Strains

Yeast strains used in this work are listed in Table 1. Strains W303-1A, SKY2939, QY364, and QY375 were obtained from Dr. Stephen Kron (University of Chicago) and JKM139 and R726 from Dr. James Haber (Brandeis University). Strains YXB1812-15 and -18 were derived from W303-1A and SKY2939, respectively, by replacing the *FUN30* open reading frame (ORF) with the *NatMX* gene cassette which confers resistance to the antibiotic nourseothricin. Strains YXB1812-24 and -25 were derived from Q364 and Q375, respectively, by replacing the *BAR1* open reading frame (ORF) with the *TRP1* gene. Note that deleting *BAR1* encoding an exported proteinase makes it significantly more efficient to arrest yeast cells in the G1 phase of the cell cycle with α-factor. Strains YXB1812-36, -38, -41, and -42 were derived from Q364 by replacing the ORFs of the *RTT107*, *DOT1*, *SLX4*, and *SAE2* genes, respectively, with *NatMX*. Strains YXB1812-37 and -39 were derived from Q365 by replacing the ORFs of *RTT107* and *DOT1*, respectively, with *NatMX*. The relevant genotype resulting from each gene replacement was verified by PCR.

### 4.2. Yeast Growth Phenotype Test

Cells were grown to the late log phase in a synthetic complete (SC) liquid medium, and 10-fold serial dilutions of the culture were spotted on test plates (SC solid medium) and were allowed to grow at 30 °C. An image of the plate was taken after 3 days of incubation.

### 4.3. SDS-PAGE and Western Blotting

Proteins were isolated from the yeast cells by TCA (tri-chloroacetic acid) extraction, as described in [40,51]. Briefly, about 4 × 10^8^ cells in the exponentially growing phase were collected and resuspended in 200 μL of a 20% TCA solution. These cells in the TCA solution were lysed using the glass bead method. Proteins were then precipitated and resuspended in 200 μL of a Laemmli buffer and were neutralized with the addition of 100 μL of 1 M Tris, pH 8.8. The protein sample was boiled for 3 min, and 10 μg of the proteins was fractionated using SDS-PAGE in 4–12% gradient gels followed by Western blotting. The blot was probed in an Odyssey blocking buffer with the goat polyclonal anti-Rad53 (yC-19: sc6749, Santa Cruz Biotechnology, Dallas, TX, USA); the rabbit polyclonal anti-H2A phospho S129 antibody (ab15083, Abcam, Cambridge, UK); the rabbit polyclonal anti-HA (H6908, Sigma-Aldrich, St. Louis, MO, USA); or the rabbit polyclonal anti-G6PD (A9521, Sigma-Aldrich), followed by the secondary antibody LI-COR IRDye 800CW of the goat polyclonal anti-rabbit IgG (H+L) 926-32211 or the LI-COR IRDye 800CW donkey anti-goat IgG (H+L) 926-32214. Imaging was performed using the LI-COR Odyssey CLx Infrared Imaging System (LI-COR Biosciences, Lincoln, NE, USA).

### 4.4. Fluorescence Activated Cell Sorting (FACS)

FACS analyses of the yeast cells were performed as described in [40,51]. Briefly, cells were grown at 30 °C to the log phase (OD_600_ = 0.6), and an aliquot was taken for FACS analysis. The rest of the cells were then arrested in the G1 phase by α-factor treatment, and an aliquot was taken for FACS analysis. The remaining cells were then released from the G1 arrest by being transferred to a fresh medium without α-factor. The resulting cell culture was then divided into two aliquots: one containing 5 μg/mL of CPT and the other without CPT. Both aliquots were incubated for 150 min. Aliquots of the cells were collected at 15 min intervals for FACS analyses. Samples of the yeast cells for FACS analysis were prepared as described [55] and were analyzed on a FACSCalibur flow cytometer (Becton, Dickinson and Company, Franklin Lakes, NJ, USA). Data analysis was performed using the FlowJo v9.9.5 software (Becton, Dickinson & Company, Franklin Lakes, NJ, USA).

## Figures and Tables

**Table 1:** Yeast strains used in this work.

#	Name	Genotype	Source/Reference
1	W303-1A	*MATa leu2-3,112 trp1-1 can1-100 ura3-1* *ade2-1 his3-11,15 rad5-535*	Ref. [44]
2	YXB1812-15	W303-1A, *fun30Δ::NatMX*	This work
3	SKY2939	W303-1A, *hta1S129A::his3MX6 hta2S129A::TRP1*	Ref. [44]
4	YXB1812-18	SKY2939, *fun30Δ::NatMX*	This work
5	QY364	JKM139, *RAD9-HA-KanMX6*	Ref. [44]
6	QY375	QY364, hta1-S129*, hta2-S129*	Ref. [44]
7	YXB1812-24	QY364, *bar1Δ::TRP1*	This work
8	YXB1812-25	QY375, *bar1Δ::TRP1*	This work
9	YXB1812-36	QY364, *rtt107Δ::NatMX*	This work
10	YXB1812-37	QY375, *rtt107Δ::NatMX*	This work
11	YXB1812-38	QY364, *do1Δ::NatMX*	This work
12	YXB1812-39	QY375, *dot1Δ::NatMX*	This work
13	YXB1812-41	QY364, *slx4Δ::NatMX*	This work
14	YXB1812-42	QY364, *sae2Δ::NatMX*	This work
15	JKM139	*MATa ho* *Δ* *hml::ADE1 hmr::ADE1 ade* *leu2–3,112 trp1::hisG lys5 ura3–52 ade3::GAL::HO1–100*	Ref. [43]
16	R726	JKM139, *hta1-S129A hta2-S129A*	Ref. [43]

## Data Availability

Data supporting the reported results are available to researchers upon request.

## Supplementary Materials

The following supporting information can be downloaded at: https://www.mdpi.com/article/10.3390/ijms25052462/s1.
