# Supplementary material for: γH2A/γH2AX Mediates DNA Damage-Specific Control of Checkpoint Signaling in Saccharomyces cerevisiae
Source: Int J Mol Sci. 2024 Feb 20;25(5):2462. doi: 10.3390/ijms25052462 (PMC10930940; doi:10.3390/ijms25052462)
Supplement: Supplementary file 1 [file ijms-25-02462-s001.zip › ijms-2829798-supplementary.pdf]

International Journal of
Molecular Sciences

MDPI

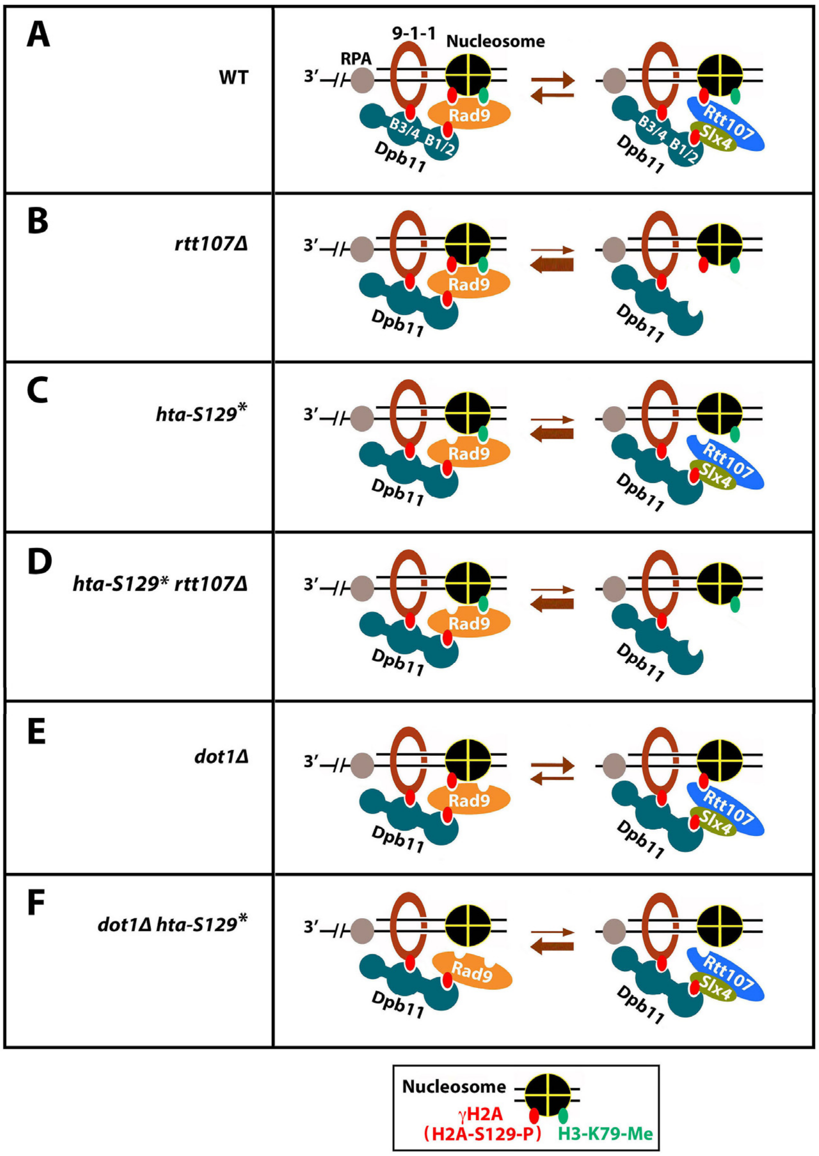

Figure S1. Illustration of the competition between Rad9 and Slx4/Rtt107 for binding γH2A and Dpb11 in various genetic backgrounds in response to DNA damage. See the text for descriptions. The genetic background considered here are (A) Wild type, (B) *rtt107Δ*, (C) *hta-S129**, (D) *hta-S129* rtt107Δ*, (E) *dot1Δ*, and (F) *dot1Δ hta-S129**.

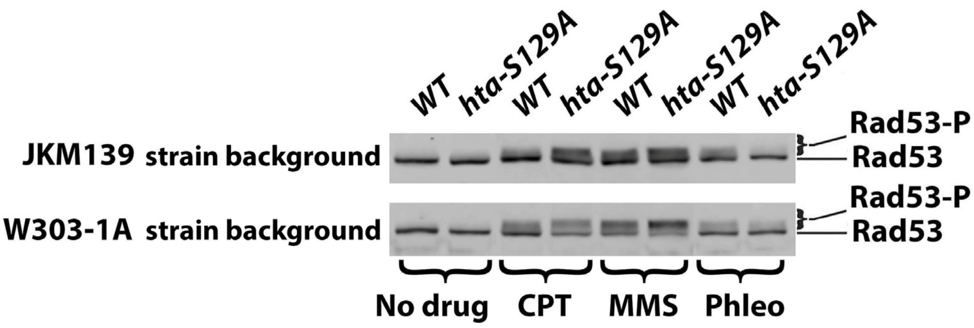

Figure S2. The effect of γH2A on DDC is DNA damage-specific. Shown is Western analysis of Rad53 from JKM139 and R726 (JKM139, *hta-S129A*) (#17 and 18 in Table 1) as well as W303-1A and SKY2939 (W303-1A, *hta-S129A*) (#1 and 3 in Table 1) treated with 5 μg/ml CPT, 0.01% MMS or 5 μg/ml phleomycin, or mock treated (No drug).
